# 2,3,5,4′-Tetrahydroxystilbene (TG1), a Novel Compound Derived from 2,3,5,4′-Tetrahydroxystilbene-2-O-β-D-glucoside (THSG), Inhibits Colorectal Cancer Progression by Inducing Ferroptosis, Apoptosis, and Autophagy

**DOI:** 10.3390/biomedicines11071798

**Published:** 2023-06-23

**Authors:** Kuei-Yen Tsai, Po-Li Wei, Cheng-Chin Lee, Precious Takondwa Makondi, Hsin-An Chen, Yao-Yuan Chang, Der-Zen Liu, Chien-Yu Huang, Yu-Jia Chang

**Affiliations:** 1 Graduate Institute of Clinical Medicine, College of Medicine, Taipei Medical University, Taipei 11031, Taiwan; leiftsai@gmail.com; 2 Department of Surgery, School of Medicine, College of Medicine, Taipei Medical University, Taipei 11031, Taiwan; poliwei@tmu.edu.tw (P.-L.W.);; 3 Division of General Surgery, Department of Surgery, Shuang Ho Hospital, Taipei Medical University, New Taipei City 235041, Taiwan; 4 Division of Colorectal Surgery, Department of Surgery, Taipei Medical University Hospital, Taipei Medical University, Taipei 11031, Taiwan; 5 Graduate Institute of Cancer Biology and Drug Discovery, Taipei Medical University, Taipei 11031, Taiwan; 6 Cancer Research Center and Translational Laboratory, Department of Medical Research, Taipei Medical University Hospital, Taipei Medical University, Taipei 11031, Taiwan; 7 Graduate Institute of Medical Sciences, College of Medicine, Taipei Medical University, Taipei 11031, Taiwan; kerwinpipi@gmail.com; 8 Kamuzu Central Hospital—National Cancer Center, Lilongwe P.O. Box 149, Malawi; khondipule@gmail.com; 9 Graduate Institute of Biomedical Materials and Tissue Engineering, College of Biomedical Engineering, Taipei Medical University, Taipei 11031, Taiwan; m225100009@tmu.edu.tw (Y.-Y.C.); tonyliu@tmu.edu.tw (D.-Z.L.); 10 Medical and Pharmaceutical Industry Technology and Development Center, New Taipei 24888, Taiwan; 11 School of Medicine, National Tsing Hua University, Hsinchu 300044, Taiwan; 12 Institute of Molecular and Cellular Biology, National Tsing Hua University, Hsinchu 300044, Taiwan; 13 Department of Pathology, Wan Fang Hospital, Taipei Medical University, Taipei 11696, Taiwan; 14 Cell Physiology and Molecular Image Research Center, Wan Fang Hospital, Taipei Medical University, Taipei 11696, Taiwan

**Keywords:** 2,3,5,4′-tetrahydroxystilbene, TG1, apoptosis, autophagy, ferroptosis, colorectal cancer

## Abstract

Background: Colorectal cancer (CRC) is one of the deadliest cancers worldwide and long-term survival is not guaranteed in metastatic disease despite current multidisciplinary therapies. A new compound 2,3,5,4′-Tetrahydroxystilbene (TG1), derived from THSG (2,3,5,4′-Tetrahydroxystilbene-2-O-β-D-Glucoside), has been developed, and its anticancer ability against CRC is verified in this study. Methods: HCT116, HT-29, and DLD-1 were treated with TG1 and the IC_50_ was measured using a sulforhodamine B assay. A Xenograft mouse model was used to monitor tumor growth. Apoptosis and autophagy, induced by TG1 in CRC cells, were examined. RNA-sequencing analysis of CRC cells treated with TG1 was performed to discover underlying pathways and mechanisms. Results: The results demonstrated that treatment with TG1 inhibited CRC proliferation in vitro and in vivo and induced apoptotic cell death, which was confirmed by Annexin V-FITC/PI staining and Western blotting. Additionally, TG1 treatment increased the level of autophagy in cells. RNA-sequencing and GSEA analyses revealed that TG1 was associated with MYC and the induction of ferroptosis. Furthermore, the ferroptosis inhibitor Bardoxolone abrogated the cytotoxic effect of TG1 in CRC cells, indicating that ferroptosis played a crucial role in TG1-induced cytotoxicity. Conclusions: These findings suggest that TG1 might be a potential and potent compound for clinical use in the treatment of CRC by inhibiting proliferation and inducing ferroptosis through the MYC pathway.

## 1. Introduction

Colorectal cancer (CRC) is considered to be one of the deadliest cancers worldwide, and its incidence is expected to increase by 60% to more than 2.2 million new cases and 1.1 million cancer deaths by 2030 [1,2]. Data from 25 population-based cancer registries from 16 European countries over the past 30 years showed improvement in 5-year relative survival in all European regions, but even the highest 5-year relative survival, which was in Switzerland, was only 65% [3,4,5]. CRC treatment options include surgery, chemotherapy, and targeted therapy [6,7]. For metastatic CRC, the initial treatment involves a combination of chemotherapeutic drugs and molecular targeted therapies [8]. However, chemoresistance is the primary reason for reduced treatment effectiveness, leading to tumor recurrence and metastasis. Targeted drugs that inhibit epidermal growth factor receptor have limited efficacy in CRC with wild type KRAS, NRAS, or BRAF genes [9]. Therefore, there is an urgent need to develop new effective or complementary therapies for metastatic CRC to improve patient outcomes.

TG1, also known as 2,3,5,4′-Tetrahydroxystilbene, is derived from THSG (2,3,5,4′-Tetrahydroxystilbene-2-O-β-D-Glucoside), the primary component of the traditional Chinese medicinal plant Polygonum multiflorum [10]. TG1 is obtained by removing the glucoside from THSG [11]. THSG has been found to possess multiple health benefits, including anti-inflammatory, antioxidant, anti-aging, and anti-atherosclerotic properties. THSG has also been shown to be beneficial in treating lipid metabolism disorders, vascular and liver fibrosis, cardiac remodeling, cardiocerebral ischemia, cognitive disorders, regenerative dentistry, neuroinflammation, hypopigmentation disease, diabetic complications, and hair growth [12,13,14,15,16,17,18,19,20,21,22,23,24,25,26]. Furthermore, THSG has been investigated for its anticancer effects in breast cancer, lung cancer, and CRC. In breast cancer, THSG combined with Adriamycin (ADM) has been shown to enhance apoptosis and reduce vascular endothelial growth factors [27]. In lung cancer, THSG treatment has been found to suppress the expression of adhesion and invasion-related factors [28]. Similarly, in CRC, THSG inhibits the metastasis of HT-29 cells by suppressing metastasis-associated proteins and NF-κB signaling [29].

A new small molecule drug called TG1 has been developed for versatile drug applications. It has been shown to have low toxicity and promote cytotoxicity in breast cancer cells when used in combination with chemotherapeutic drugs [30]. However, until now, the role of TG1 in inducing cytotoxic effects on CRC and the underlying mechanisms and downstream signaling have been unknown. In this study, TG1 was found to be effective in treating CRC through in vitro and in vivo experiments. The underlying mechanisms involved ferroptosis-related gene expression, which was responsible for the anticancer effect of TG1. Pre-treatment with a ferroptosis inhibitor was found to increase cell survival rate in TG1-treated cells. In conclusion, TG1 exhibits anticancer effects via the ferroptosis pathway, making it a promising alternative treatment option for CRC.

## 2. Materials and Methods

### 2.1. Production of TG1 (2,3,5,4′-Tetrahydroxystilbene)

In a previous study, THSG was extracted from Polygonum multiflorum using a 60% methanol solution for one day [11]. The residues were filtered, extracted twice with 60% methanol, and concentrated with a rotary evaporator. The aqueous solution was chromatographed on a Diaion HP-20 column (30 cm id × 90 cm) using H_2_O, 50% MeOH, and 100% MeOH as eluents. The 50% MeOH eluate was further chromatographed over an RH-18 column (10 cm id × 60 cm) eluted with 0.05% trifluoroacetic acid-CH_3_CN (82:18) to obtain THSG. To synthesize TG1, a solution of THSG (2.06 g, 5.62 mmol) was added to EtOH (50 mL) containing 1.0 N HCl (70 mL), refluxed for 14 h, and then extracted with ether. The organic layer was dried, concentrated, and purified by column chromatography (silica gel; DCM/MeOH = 16/1) to yield TG1 as a brown solid. TG1 was further recrystallized with CHCl_3_ to produce brown-green powder (400 mg, 32% yield). The compound structure of TG1 was determined by analyzing its ^1^H and ^13^C nuclear magnetic resonance (NMR) spectra using a Bruker Avance DRX 500 MHz instrument from Bruker Corp. (Billerica, MA, USA).

### 2.2. Chemicals, Reagents, and Cell Culture

Human colon adenocarcinoma cell lines including DLD-1 (CCL-221), HT-29 (HTB-38), and HCT116 (CCL-247) were obtained from American Type Culture Collection (ATCC, Rockville, MD, USA). All cells were cultured in RPMI 1640 Medium supplemented with 10% fetal bovine serum (FBS) (SAFC Biosciences, Lenexa, Kansas, USA) and 1% penicillin/streptomycin containing 100 IU/mL of penicillin and 100 μg/mL of streptomycin at 37 °C in 5% CO_2_ in a humidified incubator. The bardoxolone methyl used in the study was purchased from SelleckChem (Catalog No. S8078).

### 2.3. Examination of Cell Viability

A sulforhodamine B (SRB) assay was used to examine cell viability. Cells were seeded into 24-well plates at a density of 2 × 10^4^ and incubated at 37 °C in a 5% CO_2_ humidified incubator overnight. Different concentrations of TG1 (0–100 μM) were added to the cells and incubated for 48 h. After incubation, the cells were fixed with 10% (wt/vol) trichloroacetic acid at 4 °C overnight and then stained with 0.4% *w*/*v* protein-bound SRB for 30 min at room temperature. The stained cells were washed twice with 1% acetic acid and air-dried overnight. The protein-bound dye was dissolved in 10 mM Tris base solution and the optical density (OD) was measured at 515 nm using a microplate reader (Bio-Rad Laboratories, Hercules, CA, USA). The baseline was defined as cells treated with control, while fold changes were calculated as the OD values of cells treated with TG1 relative to the baseline.

### 2.4. Annexin V-FITC/Propidium Iodide (PI) Assay

A commercial apoptosis detection kit (Oncogene Research Products, Calbiochem, MA, USA) was used to perform the annexin V assay. 5 × 10^5^ cells in 500 µL of culture medium were added with 10 µL of media binding reagent and 1.25 µL of annexin V (200 µg/mL) conjugated to FITC, and then incubated for 15 min at room temperature in the dark. The suspension was centrifuged at 1000× *g* for 5 min at room temperature and the pellet was resuspended in 500 µL of 1× binding buffer with 10 µL of propidium iodide. The suspensions were placed on ice away from light and immediately analyzed by flow cytometry. Twenty thousand cells were analyzed on the FacScan cytometer under low flow conditions. Green and red fluorescence were collected using FL1 and FL2 filters, respectively, and displayed in logarithmic amplification. Electronic compensation (subtraction of 25% of FL-2 from FL-1) was required for the annexin V/PI assay. The data were analyzed using LYSYSTM II software (Becton Dickinson, Mountain View, CA).

### 2.5. Animal Model

Severe combined immunodeficient (SCID) mice were randomly divided into experimental and control groups (n = 8 per group). The mice were inoculated with 1 × 10^6^ HT-29 cells in the right dorsal subcutaneous tissue. Tumor size was measured twice a week using the following formula: tumor volume (mm^3^) = length × (width^2^)/2. After tumor volume reached 250 mm^3^, the mice were divided into five groups randomly (saline, 10 mg/kg irinotecan, 10–30 mg/kg TG1; 7 mice per group). The mice were treated with irinotecan or TG1 through the tail vein twice a week for 2 weeks. All animal use protocols were approved by the Institutional Animal Care and Use Committee of Taipei Medical University (LAC-2018-0279).

### 2.6. Autophagy Assay by the FlexiCyte Protocol

For autophagy analysis, HCT116 or DLD-1 cells (2.4 × 10^5^) were seeded into six-well plates and allowed to attach overnight. Next, cells were treated with TG1 or vehicle control for 24 h. Following treatment, cells were collected and stained with fluorescent dyes from the CYTO-ID Autophagy Detection Kit (ENZ-51031) in accordance with the manufacturer’s protocol to detect autophagic vacuoles, which include pre-autophagosomes, autophagosomes, and autolysosomes. Harvested cells were stained with Hoechst-33342 to detect the total cell population by staining nuclei. Cells were incubated at 37 °C for 15 min on a heating block. The fluorescence intensity and number were measured using the NucleoCounter NC-3000 system (ChemoMitec A/S, Allerod, Denmark).

### 2.7. Reverse Transcription Polymerase Chain Reaction (RT-PCR) and Quantitative RT-PCR Analysis

For total RNA extraction from cultured cells, TRIZOL reagent (Invitrogen Life Technologies, Carlsbad, CA, USA) was utilized. To synthesize cDNA, 8 μg of total RNA was subjected to RT reaction in a 20-μL reaction volume by using a cDNA Synthesis Kit (Invitrogen Life Technologies) as per the manufacturer’s instructions. Real-time PCR was performed using ABI SYBR Green Master Mix (Thermo Fisher Scientific, Waltham, MA, USA) on ABI 7500 FAST TM. Each sample was analyzed in triplicate, and the experiment was repeated three times. The expression levels of target genes were normalized to GAPDH.

### 2.8. Protein Extraction and Western Blot Analysis

To extract proteins from CRC cell lines, 1 × 10^6^ cells were seeded in 10 cm culture dishes. After 48 h of treatment with TG1 or the vehicle, cell lysates were separated using sodium dodecyl sulfate polyacrylamide gel electrophoresis (SDS-PAGE) and transferred onto a polyvinylidene fluoride membrane (GE Healthcare, Piscataway, NJ, USA) for antibody blotting. The membranes were incubated with primary antibodies against BCL-2, BAX, Mcl-1, cleaved (c)-caspase-3, c-PARP, Akt, phosphor (p)-Akt, LC3B-I, LC3B-II, Beclin-1, and p62 at 4 °C overnight and then probed with the respective secondary antibody. Protein bands were visualized using an enhanced chemiluminescence reagent (GE Healthcare Piscataway, NJ, USA), and detected with the VersaDoc 5000 system (Bio-Rad Laboratories, Hercules, CA, USA).

### 2.9. RNA-Sequencing Analysis

Total RNA was isolated from the CRC cells using Trizol (Invitrogen) according to the manufacturer’s protocol. RNA-sequencing was performed by Biotools biotech Co., Ltd. (Taiwan). Briefly, RNA was purified by the removal of ribosomal RNA using EpicentreRibo-Zero rRNA Removal Kit (Illumina, San Diego, California, USA) and then subjected to cDNA synthesis followed by adaptor ligation and enrichment according to the instructions of NEBNext^®^ Ultra™ RNA Library Prep Kit for Illumina (NEB, Ipswich, MA, USA). The purified library products were evaluated using Illumina NovaSeq 6000 (paired-end 150 bp). The raw reads generated from sequencing were subjected to quality filtering using Trimmomatic to obtain clean reads. Clean reads were aligned to the reference genome using HISAT2, and the raw read counts for each gene were calculated using feature Counts. The expression levels were normalized using RLE/TMM/FPKM methods. Differentially expressed genes were determined based on a cutoff of a two-fold change with an adjusted *p*-value < 0.05.

### 2.10. Statistical Analysis

The data was analyzed using the statistical functions of Microsoft Excel. Results are presented as mean ± standard deviation (SD) from a minimum of three independent experiments. For IC_50_ experiments, data was analyzed using one-way ANOVA or a two-tailed unpaired *t*-test for comparison. A *p*-value of less than 0.05 was considered statistically significant (*, *p* < 0.05; **, *p* < 0.01).

## 3. Results

### 3.1. TG1 Possesses Dose-Dependent Cytotoxicity on Colorectal Cancer Cells

**Figure 1:**
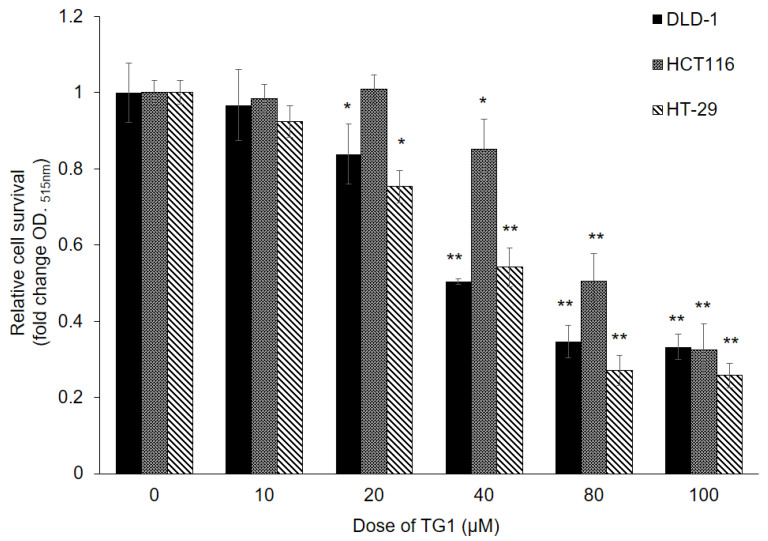
TG1 exhibited cytotoxicity in colorectal cancer cells. After treatment with TG1 at different doses (0 to 100 μM), the relative cell survival rate was determined through the SRB colorimetric assay. TG1 resulted in reduced cell survival in DLD-1, HCT116, and HT-29 cells in a dose-dependent manner compared with the survival of the control-treated cells (* *p* < 0.05, ** *p* < 0.01).

In order to investigate the potential anticancer effect of TG1 on CRC cells, an SRB assay was conducted on three different CRC cell lines (DLD-1, HCT116, and HT-29). The cells were exposed to varying concentrations of TG1 (ranging from 0–100 μM) for a duration of 48 h. The results depicted in Figure 1 indicate that protein synthesis ability, representing cell viability of CRC cells, decreased in a dose-dependent manner following treatment with TG1. The IC_50_ values for DLD-1, HCT116, and HT-29 cells were 40 μM, 80 μM, and 45 μM, respectively, suggesting that TG1 may possess the ability to inhibit the proliferation of CRC cells and exhibit potential anticancer properties.

### 3.2. TG1 Treatment Suppresses CRC Progression in a Xenograft Mouse Model

**Figure 2:**
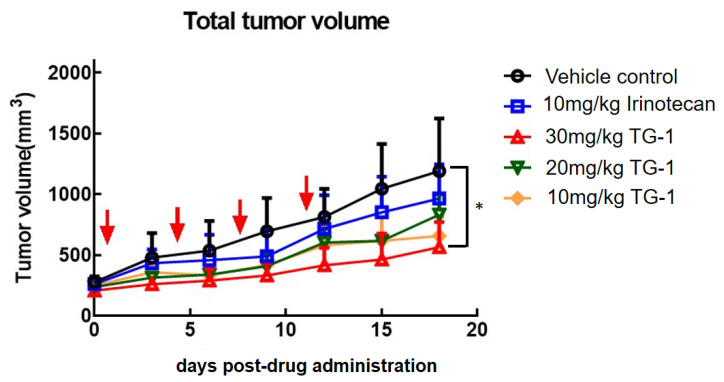
Inhibition of tumor progression by TG1 and irinotecan in xenograft model. Growth of tumor volume was inhibited effectively by TG1, while dose-dependent inhibitory effects of TG1 were demonstrated. Arrows indicate time points when drugs were administered. In addition, the inhibitory effect of TG1 was better than that of irinotecan, which is a common chemotherapeutic agent in clinical use. * *p* < 0.05.

To explore the potential therapeutic benefits of TG1, a xenograft model using HT-29 cells was established by implanting them into the right dorsal subcutaneous layer of mice. Once the estimated tumor volume reached 250 mm^3^, TG1 was administered. The mice were treated with irinotecan or TG1 through the tail vein twice a week for 2 weeks. The findings, illustrated in Figure 2, indicated that treatment with TG1 significantly reduced tumor volume when compared to the control group in a dose-dependent manner. In addition, TG1 shows a better anticancer effect than irinotecan. Those results indicate that TG1 has a strong inhibitory effect on tumor progression. These results suggest that TG1 may have promising anticancer properties in the context of a CRC xenograft model.

### 3.3. CRC Cells Apoptosis Is Induced by TG1

**Figure 3:**
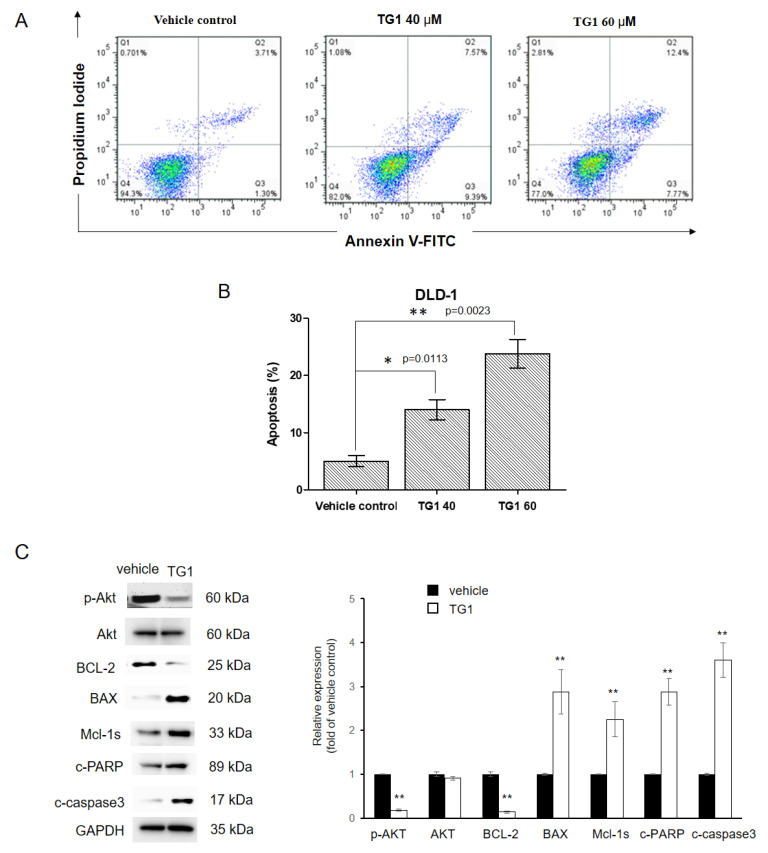
Treatment with TG1 induced apoptosis in colorectal cancer cells. DLD-1 cell apoptosis was analyzed using Annexin V-FITC/propidium iodide staining and FACS analysis after being treated with 40 and 60 μM TG1 (**A**,**B**). Protein levels of apoptosis-related genes were determined by Western blotting (**C**). DLD-1 cells treated with 60 μM TG1 exhibited increased levels of BAX, Mcl-1s, c-PARP, and c-caspase 3, whereas levels of p-Akt and BCL-2 were decreased. All experiments were performed at least three times independently (* *p* < 0.05, ** *p* < 0.01).

Apoptosis has been proven to play an important role in tumor suppression [31]. It is currently unknown whether the cytotoxic effects of TG1 on CRC cells are correlated with the induction of apoptosis. To investigate this, the apoptotic potential of TG1 was assessed using an Annexin V/propidium iodide double-staining assay. Flow cytometry analysis revealed that TG1 treatment significantly increased the ratio of apoptosis in CRC cells when compared to the vehicle control (Figure 3A,B). Specifically, following treatment with 40 and 60 μM of TG1, the percentage of apoptotic cells increased from 5.01% to 16.96% and 20.17%, respectively. Additionally, Western blot analysis demonstrated upregulation of various pro-apoptotic proteins including c-PARP, c-caspase 3, BAX, and Mcl-1s, and downregulation of the anti-apoptotic proteins Bcl-2 and p-Akt in CRC cells treated with TG1, as compared to the control group (Figure 3C,D). Taken together, these findings suggest that the ability of TG1 to suppress CRC cells is regulated by inducing apoptosis.

### 3.4. TG1 Treatment Causes an Induction of Autophagy Process

**Figure 4:**
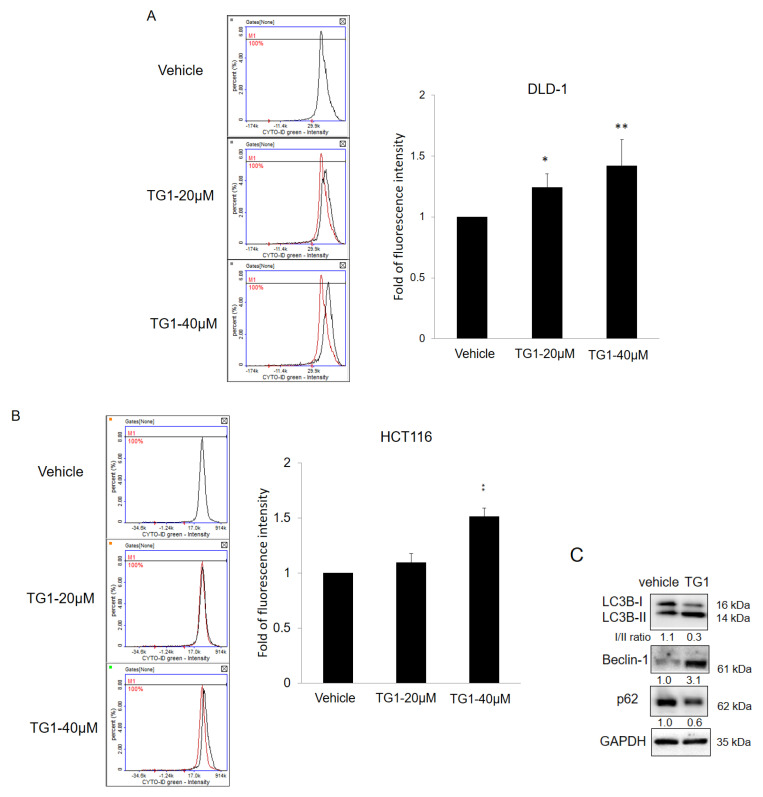
Treatment with TG1 induced autophagy in colorectal cancer cells. DLD-1 and HCT116 cells were treated with TG1 at 20 μM and 40 μM for 48 h, and autophagic vacuoles and autophagic flux were detected using a Cyto-ID Autophagy Detection Kit, which demonstrated autophagy was increased after the treatment (**A**,**B**). Relative levels of autophagy-related proteins were obtained via Western blot analysis, which showed autophagy was induced after treatment with TG1 (**C**) (* *p* < 0.05, ** *p* < 0.01).

Autophagy, a process that involves the selective degradation of cellular components, is an essential mechanism that plays dual roles in cancer progression and suppression, which could elicit tumor cell apoptosis in cooperation with anticancer drugs [32]. To determine whether TG1 induces the activation of autophagic processes, a Cyto-ID Autophagy Detection Kit was used to detect autophagic vacuoles and autophagic flux. Levels of autophagy were upregulated in CRC cells treated with TG1 compared with those treated with the vehicle control (Figure 4A,B). Furthermore, Western blot analysis was performed to verify the expression of autophagy-related proteins. Beclin-1 was upregulated, and the conversion of LC3B-I to LC3B- II was increased significantly by treatment with TG1, while the level of p62 was decreased correspondingly in the process (Figure 4C). These data suggest that treatment with TG1 induces autophagy process in CRC cells.

### 3.5. Analysis of Gene Expression Changes in TG1 Treatment

**Figure 5:**
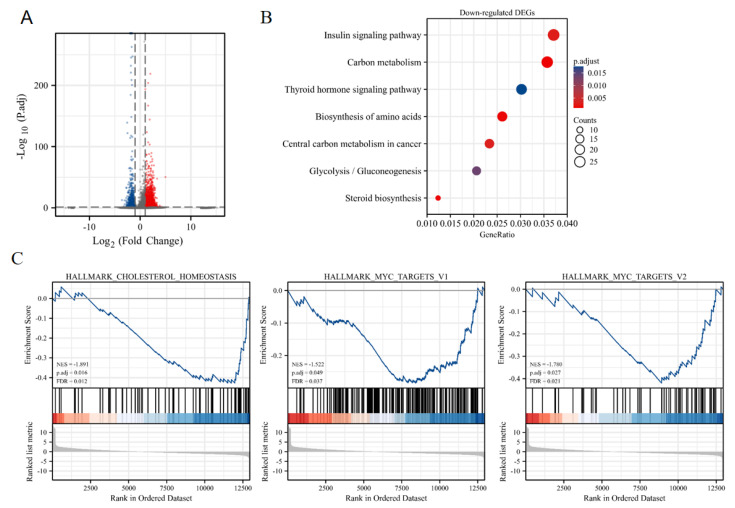
Pathway enrichment analysis of colorectal cancer cells treated with TG1. (**A**) Identification of DEGs between TG1 treatment and control group based on RNA-sequencing data. Volcano plot displaying the significant 1837 upregulated DEGs (red) and 1437 downregulated DEGs (blue). (**B**) KEGG pathway enrichment analysis of downregulated DEGs between control and TG1 treated group from RNA-seq data. Bubble plot showing seven metabolic associated pathways (*p*-value < 0.05, q-value < 0.25). (**C**) GSEA analysis based on the log_2_ fold changes of protein-coding genes between control and TG1 treated group.

To identify the pathways involved in TG1-induced cytotoxicity, RNA-sequencing (RNA-seq) analysis was used to explore differentially expressed genes (DEGs) in HCT116 cells between TG1 treatment (60 μM) and control. A total of 1837 genes were significantly upregulated after 48 h of TG1 treatment, while 1437 genes were significantly downregulated (Figure 5A). To understand the inhibitory effect of TG1, we performed KEGG pathway analysis on downregulated DEGs. We found seven metabolism-related pathways, including insulin signaling pathway, carbon metabolism, thyroid hormone signaling pathway, biosynthesis of amino acids, central carbon metabolism in cancer, glycolysis/gluconeogenesis, and steroid biosynthesis (Figure 5B). Furthermore, we conducted Gene Set Enrichment Analysis (GSEA) of DEGs and found that the downregulated DEGs were enriched in HALLMARK_CHOLESTEROL_HOMEOSTASIS, HALLMARK_MYC_TARGETS_V1 and HALLMARK_MYC_TARGETS_V2 (Figure 5C), suggesting that TG1 may regulate cancer cell metabolism and influence cell survival by inhibiting MYC and its downstream effector molecules.

### 3.6. TG1 Treatment Regulates Expression of Ferroptosis-Related Genes

**Figure 6:**
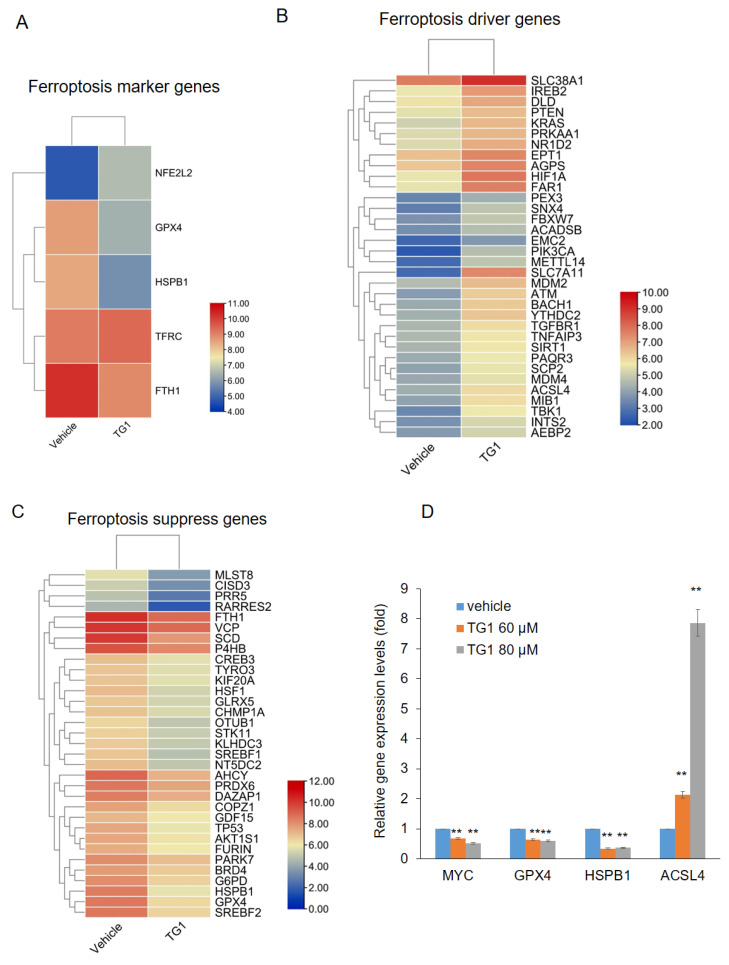
Differentially expressed ferroptosis-related genes between the control group and the TG1 treatment group. (**A**) Five marker genes of ferroptosis-related genes. (**B**) 34 driver genes of ferroptosis-related genes. (**C**) 33 suppressor genes of ferroptosis-related genes. (**D**) The expression of MYC, GPX4, HSPB1, and ACSL4 was validated through RT-qPCR analysis (** *p* < 0.01).

Ferroptosis is a recently discovered novel programmed cell death characterized by the accumulation of iron-dependent lipid peroxides, while its morphological and genetical forms are distinct from other regulated cell deaths, such as apoptosis. More and more studies have found that the induction of ferroptosis can successfully achieve cytotoxicity in CRC cells resistant to other cell death modes [26]. Therefore, targeting ferroptosis is considered a new therapeutic point for the treatment of colorectal cancer. To investigate whether TG1 affects ferroptosis, we crossed the DGEs in the TG1 treatment with ferroptosis-related genes from the FerrDb database (http://www.zhounan.org/ferrdb/, accessed on 4 May 2023). The intersection of two gene sets contained 5 marker genes, 34 driver genes and 33 suppressor genes of ferroptosis. Of these marker genes, ferritin heavy chain 1 (FTH1), glutathione peroxidase 4 (GPX4), and heat-shock protein family B member 1 (HSPB1) play roles of suppressor in ferroptosis, and our RNA-seq results showed these genes were downregulated noticeably after treatment of TG1 (Figure 6A). Ferroptosis driver genes were upregulated in the TG1 treated group compared with the vehicle control (Figure 6B), while treatment with TG1 downregulated the ferroptosis suppressor genes (Figure 6C). To verify the results of RNA sequencing, we performed RT-qPCR to examine gene expression after treatment of DLD-1 cells with TG1 (60 μM and 80 μM) and found that the expression of MYC was decreased in accordance with GSEA analysis (Figure 6D). Of the genes related to ferroptosis, the expression of GPX4 and HSPB1, two marker genes with ferroptosis-suppressing effects, were reduced, whereas the expression of acyl-CoA synthetase long chain family member 4 (ACSL4) was increased (Figure 6D). Taken together, these results exhibit TG1 induces ferroptosis in CRC cells via upregulating ferroptosis driver genes and downregulating ferroptosis suppressor genes.

### 3.7. Prevention of TG1-Induced Cancer Cell Death by Inhibiting Ferroptosis

**Figure 7:**
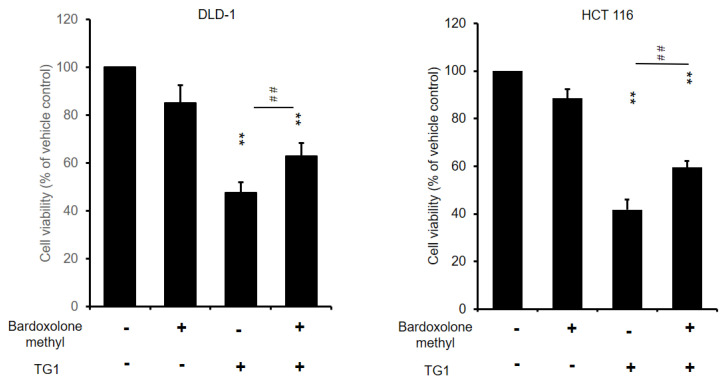
Blocking ferroptosis rescued TG1-induced cytotoxicity in colorectal cancer cells. The viability of CRC cells treated with TG1, in the presence and absence of bardoxolone methyl, was determined via SRB colorimetric assay (** *p* < 0.01, ## *p* < 0.01).

In order to explore the role of ferroptosis in the cytotoxicity of TG1 on CRC cells, a ferroptosis inhibitor, bardoxolone, was used in combination with TG1. Bardoxolone methyl is known to inhibit ferroptosis without exhibiting any antioxidant activity [33]. To investigate the regulation of ferroptosis in TG1-induced cancer cell cytotoxicity, DLD-1 and HCT116 cells were treated with bardoxolone in combination with TG1 at concentrations of 40 and 60 μM. Treatment with bardoxolone alone did not affect the viability of DLD-1 cells, but co-treatment with bardoxolone rescued the TG1-induced cytotoxicity (Figure 7). Similarly, bardoxolone treatment consistently exhibited a rescue effect on TG1-induced cancer cell death in HCT116 cells (Figure 7). These findings suggest that the cytotoxic effect of TG1 on CRC cells is mainly regulated by ferroptosis.

## 4. Discussion

CRC is highly prevalent all over the world, but the long-term survival rate for metastatic disease remains unsatisfactory. Chemo-resistance is a major cause of cancer recurrence in CRC patients. Discovery of new drugs is urgently needed in order to improve the cancer prognosis. Based on our results, TG1 had notable cytotoxic effects in CRC cells, as well as significant anti-tumor effects in mouse model. Treatment with TG1 induced CRC cell apoptosis and autophagy, while RNA-sequencing analysis as well as RT-qPCR results showed TG1 also induced ferroptosis. Further, inhibition of ferroptosis prevented TG1-induced cancer cell death. These results indicate that TG1 is a potent anticancer compound, and its effect may be achieved through the mechanism of induction of ferroptosis.

TG1 is structurally like 2,3,5,4′-tetrahydroxystilbene-2-O-β-D-glucoside (THSG) and resveratrol (3,4′,5-trihydroxystilbene). Resveratrol, a natural stilbene and a non-flavonoid polyphenol, has been reported to be able to reverse multidrug resistance and sensitize cancer cells to standard chemotherapeutic agents [34,35]. In breast cancer, a combination of THSG and ADM treatment increased the cell apoptosis by increasing apoptosis related markers including Bax/Bcl-2 and cleaved caspase-3/caspase-3 and decreasing vascular endothelial growth factor/phosphatidylinositol 3-kinase/Akt protein expression [27]. A previous study reported that THSG suppressed the CRC metastasis by decreasing NF-kappaB pathway signaling [29]. THSG and resveratrol are reported to have anti-inflammatory, anti-oxidative effects [36], and multiple pharmacological uses in cancers [27,28].

Over the past three decades, one-third of new FDA approved drugs were derived from natural products, and 74% were used for anticancer therapies [37]. Natural products are important sources of innovative drug development [38], but clinical applications are limited due to poor bioavailability or severe toxicity [39]. TG1, modified from 2,3,5,4′-tetrahydroxystilbene-2-O-β-D-glucoside (THSG) via deglucosylation, has been proven to have low toxicity in vitro and in vivo, while this modification may improve the drug potency via the reduction of the drug efflux effect [30]. Further, our results found that the cytotoxic effect of TG1 on CRC cells does not only come from inducing apoptosis, but more importantly, it regulates the induction of ferroptosis. Cancer cells adapt their pathways for survival during stress conditions, and a good therapeutic agent achieves its cytotoxicity via concurrently different mechanisms, suggesting TG1 potential for further clinical applications.

Autophagy is a process involving selective degradation of cellular components, and it is generally considered to play roles in tumor suppression, as dysfunction of autophagy has been reported to cause increased oxidative stress as well as tumorigenesis [40]. Several reports have shown autophagy results in ferroptosis through degrading ferritin in tumor cells as well as increasing oxidative damage [41,42], while our results showed that TG1 treatment induces autophagy and ferroptosis consistently. On the other hand, MYC, a proto-oncogene, is often constitutively upregulated in cancers of the cervix, colon, breast, lung, and stomach [43]. Mutation of c-myc in CRC is an independent negative survival predictor, and c-myc could enhance tumor proliferation, metastasis, and triggering chemoresistance in CRC [44]. The current study revealed that treatment with TG1 leads to downregulation of MYC via GSEA analysis and RT-qPCR assay, indicating that TG1 has potency of becoming a promising therapeutic agent in CRC.

Ferroptosis, a type of cell death dependent on iron overload, ROS accumulation, and lipid peroxidation, gains more and more attention in the treatment strategy in oncology [45]. Ferroptosis-related genes, GPX4 and FTH1, were reported upregulated in patients undergoing neoadjuvant chemoradiotherapy and associated with recurrence and poor prognosis in CRC, while CRC cells were sensitive to GPX4 inhibitor [46]. Our results also confirmed that treatment with TG1 downregulates expression of GPX4 in RNA-seq and RT-qPCR assays. Another marker gene of ferroptosis, HSPB1 (also known as heat-shock protein 27), was reported associated with 5-FU resistance in human CRC cells, and inhibition of HSPB1 reduced 5-FU resistance [47]. A study regarding glioblastoma also showed that a knockdown of HSPB1 evidently induced ferroptosis and enhanced ACSL4 stability via reducing SUMOylation of ACSL4 [48]. The current study consistently demonstrated that TG1 treatment achieved inhibition of HSPB1 as well as upregulation of ACSL4. However, ACSL4 is regarded as playing complex roles in tumor promotion and tumor suppression in different cancer types, while it plays important parts in lipid metabolism reprogramming, which shows different effects on tumors [49]. The exact role of ACSL4 should be investigated.

## 5. Conclusions

In conclusion, our results elucidate that TG1 exhibits a cytotoxic effect on CRC cells in vitro and suppresses tumor growth in vivo. Treatment with TG1 not only inhibits expression of MYC but also induces autophagy in CRC cells, accompanied with apoptosis and ferroptosis. Expression of ferroptosis-related genes, including FTH1, GPX4, HSPB1, and ACSL4, is significantly changed after TG1 treatment. Further, the cytotoxicity of TG1 is reversed by a ferroptosis inhibitor; this result confirms the mechanism of TG1. Taken together, these findings suggest that TG1 may be a promising and potent treatment alternative for colorectal cancer, and that TG1 is worthy of further investigation and application.

## Data Availability

The dataset supporting the conclusions of this article is included within the article.
